# Eurhythmics and Health

**Published:** 1920-09-11

**Authors:** 


### THE PUBLIC WELL-BEING.

### Eurhythmics and Health.

### TRAINING MIND AND BODY BY RHYTHM.

The summer vacation course for eurhythmics, which has just been held at Oxford, is in itself an indication of the growing public interest in this interesting and delightful form of education, which combines the physical with the mental in a subtle and extraordinary manner. What are eurhythmics and what is the use of them? Sir Michael Sadler, Vice-Chancellor of the Leeds University, who was one of the first people in this country to recognise the potential value of eurythmics in education, has done much to enlighten the public in the subject and to arouse the enthusiasm of teachers. Monsieur Jaques-Dalcroze, in introducing the system, "re-opened a door which had long been closed." He has rediscovered, says Sir Michael Sadler, one of the secrets of Greek education.

His efforts began in the training of students of music. But it was quickly seen that his ideas had even a wider application. His experience suggested the possibility of a very close combination of the intellectual and artistic elements in elementary and secondary education. His teaching requires from the pupils a sustained and careful attention. It is a severe, though not exhausting, intellectual exercise. At the same time, it trains the sense of form and rhythm, the capacity of analysing musical structure, and the power of expressing rhythm through harmonious movement. "Its educational value for children, its applicability to their needs, the pleasure which they take in its exercises have been conclusively proved. Admirable for those who are making a special study of music, it has also shown its value as a factor in general education." Monsieur Jaques-Dalcroze writes: "My system teaches how to estimate the duration of movements of the body, to graduate such movements according to the degree of tension and relaxation, and, by interplay and counterplay of the muscles, to make feeling for rhythm a physical experience. By developing our sense of rhythm we develop all our faculties, give them free play, and thus bring our whole being to the highest state of efficiency."

### Examples of the Exercises.

The exercises may, on the whole, be divided into two groups; we may call them, remarks Sir Michael Sadler, exercises of control and exercises of interpretation. As an example of the exercises of control, take the following: The pupils march round the room in time to the music, and at the teacher's command immediately take one step backwards and then go on again. This exercise may be fairly easy if the speed is slow and the commands come at long intervals, but at a moderately quick speed, and when the commands come in rapid succession it will be found bewilderingly difficult to accomplish. Here is a similar exercise, more difficult because it contains two factors instead of one: The pupils march and clap their hands in time to the music; when the teacher says "Hand" the pupils omit one clap, when the teacher says "Foot" they omit one step. In this case also, where it is taken quickly and the commands are alternated and repeated with rapidity, a beginner has a sensation of mental confusion and lack of physical control which results in a curious feeling of helplessness, to which is often added exasperation at being unable to accomplish such apparently simple movements. Besides these exercises, the object of which is to enable the will to check or alter a movement with rapidity and certainty, there are exercises to train different parts of the body to move independently of each other. For instance, the pupil beats four-time—somewhat in the fashion of a conductor, but with both arms; at the teacher's command, the left arm stops for one beat while the right arm goes on, but on the next beat the left continues so that both arms are then again beating four-time, but the right arm is a beat ahead of the left. Then, again, the right arm may beat four, while the left beats three, the head two, and the feet five. These exercises, unlike the first described, sound more complicated and difficult than they are. In a very short time they become automatic, and can be performed by children without any feeling of strain.

We then come to the exercises of interpretation. In order to make these clear, it must first be explained that in general the arms are used for beating time, while the feet take one step for every note played. For instance, in a bar consisting of one crotchet and two quavers the arms would make two regular movements because the bar is in two-four time, while the feet would make three steps, the last two twice as quick as the first. Many exercises merely consist in the pupil listening to a bar, or series of bars, played by the teacher, and afterwards *realising* them, as it is called, with these arm and leg movements. But not only the time and actual notes are to be shown by the body—every shade in the music, the change from staccato to legato, the crescendos and diminuendos, the accelerandos and ritenutos, must all be instantly rendered by physical movement. This will perhaps give some idea of what we are doing when we are doing rhythmic movement.

### Effect on Mind and Body.

We have given this rather detailed explanation of some of the methods employed because it is almost impossible to describe them in general terms. But a written description of the exercises is totally inadequate to convey a just impression of their remarkable beauty and the obvious effect on those who take part in them. The writer has seen many demonstrations of eurhythmics in the schools by children, and on educational platforms by adults; and he will never forget the grace and

**THE PUBLIC WELL-BEING**—(*continued*).

charm of the movements, the mysteriously perfected sense of rhythm which even little children appeared to have developed, the beautifully free play of all the muscles of those who took part, the picture which they presented of abounding health, and the intense joy which was revealed in their faces, and in every bodily expression, in carrying out these apparently complicated but carefully graduated movements of physical rhythm. School-teachers have told him that eurhythmics have brightened the faculties of dull children almost beyond belief, besides, in some instances, bringing to light hidden physical weaknesses which would otherwise have remained undiscovered, and in many others definitely improving the physique and figure of undersized and ill-formed pupils.

Neurasthenia is often nothing else than intellectual confusion produced (in the words of the originator of the method) by the inability of the nervous system to obtain from the muscular system regular obedience to the order of the brain. "Training the nerve centres, establishing order in the organism, is the only remedy for intellectual perversion produced by lack of will-power and by the incomplete subjection of body to mind. Unable to obtain physical realisation of its ideas, the brain amuses itself in forming images without hope of realising them, drops the real for the unreal, and substitutes vain and vague speculation for the free and healthy action of mind and body." The first result of a thorough rhythmic training is that the pupil sees clearly in himself what he really is, and obtains from his powers all the advantage possible. That seems to be a sound view of the psychological aspect of the matter, and one which should attract the attention of all educationalists and assure to education "by and for rhythm an important place in general culture." It is no mere cult of a few cranks. It supplies, as Sir Michael Sadler once said, a human need felt by nearly everyone, though often, maybe, unconsciously—the need of expressing musical motion by movement and the need of expressing in a group the common motion of the group. It is a healthy sign that all over the country those responsible for the school curricula are beginning to see the possibilities of eurhythmics and introducing them in some form or other into the school plan. We believe several enterprising elementary school-teachers are to be numbered among these, although the Board of Education has not yet authoritatively adopted the system.

